# Synthesis and characterization of Ag@AgCl-reinforced cellulose composites with enhanced antibacterial and photocatalytic degradation properties

**DOI:** 10.1038/s41598-021-82447-2

**Published:** 2021-02-09

**Authors:** Yan-Yan Dong, Ya-Hong Zhu, Ming-Guo Ma, Qi Liu, Wen-Qing He

**Affiliations:** 1 Institute of Environment and Sustainable Development in Agriculture, Chinese Academy of Agricultural Sciences, Beijing, 100081 People’s Republic of China; 2 College of Materials Science and Technology, Beijing Forestry University, Beijing, 100083 People’s Republic of China; 3 College of Forestry, Northwest A&F University, Yangling, 712100 Shaanxi People’s Republic of China

**Keywords:** Environmental sciences, Engineering, Materials science, Nanoscience and technology

## Abstract

In the present work, Ag@AgCl-reinforced cellulose composites with enhanced antibacterial and photocatalytic degradation properties were successfully synthesized via oil bath heating method. During the process, zinc chloride (ZnCl_2_) solution was used as both Cl^−^ resource to form AgCl and the solvent to dissolve cellulose. The samples were synthesized with different temperatures, times, and concentrations of ZnCl_2_ solution. The morphology, microstructure and phase of the as-prepared samples were analyzed with X-ray powder diffraction (XRD), fourier transform infrared (FTIR) spectrometry, scanning electron microscopy (SEM), transmission electron microscopy (TEM), high-resolution transmission electron microscopy (HR-TEM), X-ray photoelectron spectroscopy (XPS), photocatalytic activity studies and inhibition zone experiments. Results showed that dye solution could be completely degraded by the materials in 1 h, and higher concentrations of ZnCl_2_ solution favored for larger inhibition zones (higher to 10.8 mm). This synthetic strategy displayed here offers more possibilities to high value-added applications of cellulose.

## Introduction

With advantages of excellent stability, outstanding photocatalytic performance, and antibacterial properties, Ag@AgX(X = Cl, Br) composites materials have attracted a lot of attentions^1–9^. Fan et al. reported the synthesis of 3D AgX/graphene aerogel (GA) composites (X = Br, Cl). According to the results, the as-prepared composites showed higher photocatalytic performance than pristine AgX did, and that the photocatalytic cycling process was facilitated just using tweezers^10^. The synthesis of Ag/AgX/SrTiO_3_ (X = Cl, Br) photocatalysts via a facile reverse microemulsion method was reported in the previous work^11^, the work showed that Ag/AgX/SrTiO_3_ (X = Cl, Br) photocatalysts exhibited higher photocatalytic activity towards the degradation of Rhodamine B and methyl orange under the visible light illumination as compared to that of Ag/AgClSrTiO_3_. Li et al. reported the successful synthesis of one-dimensional (1D) Z-scheme AgCO_3_/Ag/AgBr ternary heterostructured nanorods via a facile reaction route, with visible-light induced reduction followed by an anion exchange process, the as-prepared samples showed excellent photostability and photoactivity^12^. The magnetic Ag/AgBr@Fe_2_O_3_ composite with core–shell structures was synthesized by a facile solvothermal method, and showed enhanced photocatalytic activity for organic pollutant degradation and antibacterium^13^. A novel bimetallic silver halide (Au/AgBr–Ag) plasmonic heterostructure as a visible light induced photocatalyst was reported to be successfully synthesized by Purbia and Paria^14^. The sunlight active Ag/AgBr Janus nanoparticles (JNPs) were reported to be successfully synthesized using non-toxic surface active ionic liquid via a new sustainable approach, the as-prepared JNPs showed not only excellent catalytic activity for photo-degradation of Rhodamine-B under sunlight but also effective reduction of 4-nitrophenol^15^. Lin et al. reported the synthesis of Ag/AgCl plasmonic cubes, which could be used as advanced visible-light photocatalysts, and showed ultrahigh activity for photodegrading dyes via a simple, facile, rapid and green technique^16^. Song et al. reported the synthesis of Ag/AgBr/AgCl hollow microspheres using template assisted synthesis process followed by calcination and light reduction method^17^.

However, there are still a lot of works that need to be done to form the metallic Ag^0^ species by photoreduction or chemical reduction methods, which usually involved in the employment of NaBH_4_, formaldehyde, polydopamine^18^, polyvinyl alcohol (PVA)^19^, and H_2_O_2_ or poly (N-vinyl-2-pyrrolidone) (PVP)^20^, all of them are hazardous or toxic reducing agents, which may pose environmental and health risks^21,22^. It is of much importance to explore a facile way to synthesize Ag@AgX(X = Cl, Br) composites with good properties via a green strategy. Cellulose, one of the most abundant resources of biomass on the earth, has a lot of outstanding features, such as biodegradability, flexibility, and renewability^23^. What is more, there are large numbers of hydroxyl groups on the surface of cellulose, which show reductive ability and could reduce noble metal ions to noble metals. The electronic feature of hydroxyl groups on the surface of cellulose can also stabilize the metal nanoparticles on their surfaces and control the growth of them^24,25^. It is reported that cellulose microgels and water-soluble cellulose acetate could be used also as reducing agent to synthesize Ag nanoparticles^26,27^. During the past few years, there are only a few works focused on this area, in the previous literature, our group has employed (nano)cellulose as substrates and reductive agents to synthesize Ag-based cellulose composites materials^28,29^.

In the present work, we focused on the green, in-situ synthesis of Ag@AgCl-reinforced cellulose composites materials, wherein no other additives were added. The as-synthesized Ag@AgCl-reinforced cellulose composites exhibited a superhigh antibacterial properties against both *Staphyloccus aureus* (*S. aureus*) and *Escherichia coli* (*E. coli*), and excellent photocatalytic degradation activity for methylene *blue* (MB) solution, suggesting that the materials could be used as effective growth inhibitors against microorganisms and extending the potential application in biomedical and water environment pollution field.

## Experimental

### Preparation of Ag@AgCl-reinforced cellulose composites

All chemicals were of analytical grade and used as received without further purification. All experiments were conducted under air atmosphere. According to the procedure reported in our previous work^4,9^, 37.14 g of ZnCl_2_ (65% w/w) was added into 20 mL of distilled water under vigorous stirring via oil bath heating method to form solution. Then, 0.50 g of microcrystalline cellulose was added into the above solution at 60 °C under vigorous stirring for 4 h to form cellulose solution. At last, 0.34 g AgNO_3_ was added into the cellulose solution and the mixed solution were kept for 1 to 3 h at 50, 60, and 70 °C, respectively. In order to further explore the influences of ZnCl_2_ concentrations on the materials, the samples were also synthesized with 30.00 g of ZnCl_2_ (60% w/w) and 46.67 g of ZnCl_2_ (70% w/w), while keeping the other situation the same. The product was separated from the solution by centrifugation, washed with water and ethanol three times, and dried at 60 °C for further characterization.

### Characterization of Ag@AgCl-reinforced cellulose composites

X-ray powder diffraction (XRD) patterns were performed in 2θ range from 10° to 70° on a Rigaku D/Max 2200-PC diffractometer with Cu Kɑ radiation (*λ* = 0.15418 nm) and graphite monochromator at ambient temperature^3^. Fourier transform infrared (FTIR) spectra of the as-synthesized Ag@AgCl-reinforced cellulose composites were taken on a Thermo Scientific Nicolet iN10 FTIR Microscope (Thermo Nicolet Corporation, Madison, WI, USA) in the range of wave number from 4000 to 675 cm^−1^. The morphologies and microstructures of hybrids were examined using scanning electron microscopy (SEM, Hitachi 3400N, accelerating voltage 15 kV), transmission electron microscopy (TEM, HT7700, accelerating voltage 120 kV), and high-resolution transmission electron microscopy (HRTEM, JEM-2100F, accelerating voltage 200 kV). All samples were Au coated prior to examination by SEM^1^. The X-ray photoelectron spectroscopy (XPS) analysis was performed on a ESCALAB 250Xi X-ray photoelectron spectroscope with a monochromatic Al K*ɑ* radiation (hν = 1486.6 eV) and the banding energies were normalized to C 1 s peak at 284.8 eV (Thermo Scientific Co., England)^9^.

### Antibacterial activity studies

Following the method mentioned in the previous paper^2^, the antibacterial activities of Ag@AgCl-reinforced cellulose composites were investigated against *E. coli* as the model Gram-negative bacteria and *S. aureus* as the model Gram-positive bacteria by the disc diffusion method. In the inhibition zone experiment, nutrient agar was poured into disposable sterilized Petri dish and solidified. Then 100 μL of *E. coli* and 100 μL of *S. aureus* were streaked over the dish and spread uniform. After that, circular pieces of the control and the test samples were gently placed on Petri dishes. This was done for both the bacterial strains (*E. coli* ATCC HB101 and *S. aureus* ATCC 25,923). The Ag@AgCl-reinforced cellulose composites were cut into a disc shape with 1.4 cm diameter, sterilized by autoclaving at 120 °C for 20 min, and placed on *E. coli*-cultured and *S. aureus*-cultured agar plates, which were then incubated at 37 °C for 24 h. Finally, the inhibition zone was monitored.

### Photocatalytic activity studies

For the photocatalytic measurements, 15 mg of Ag@AgCl-reinforced cellulose were dispersed in 50 mL of methylene blue (MB) solution (20 mgL^−1^). Prior to irradiation, the suspension was magnetically stirred for 20 min in dark to make sure the establishment of an adsorption/desorption equilibrium. After that, the suspension was exposed to 600 W metal mercury lamp for different times in the time range of 0 to 1 h. About 5 mL of suspension was centrifuged with 8000 rpm to remove the catalyst and then the supernatant was measured absorbance tested with an UV–Vis spectrophotometer.

## Results and discussion

**Figure 1:**
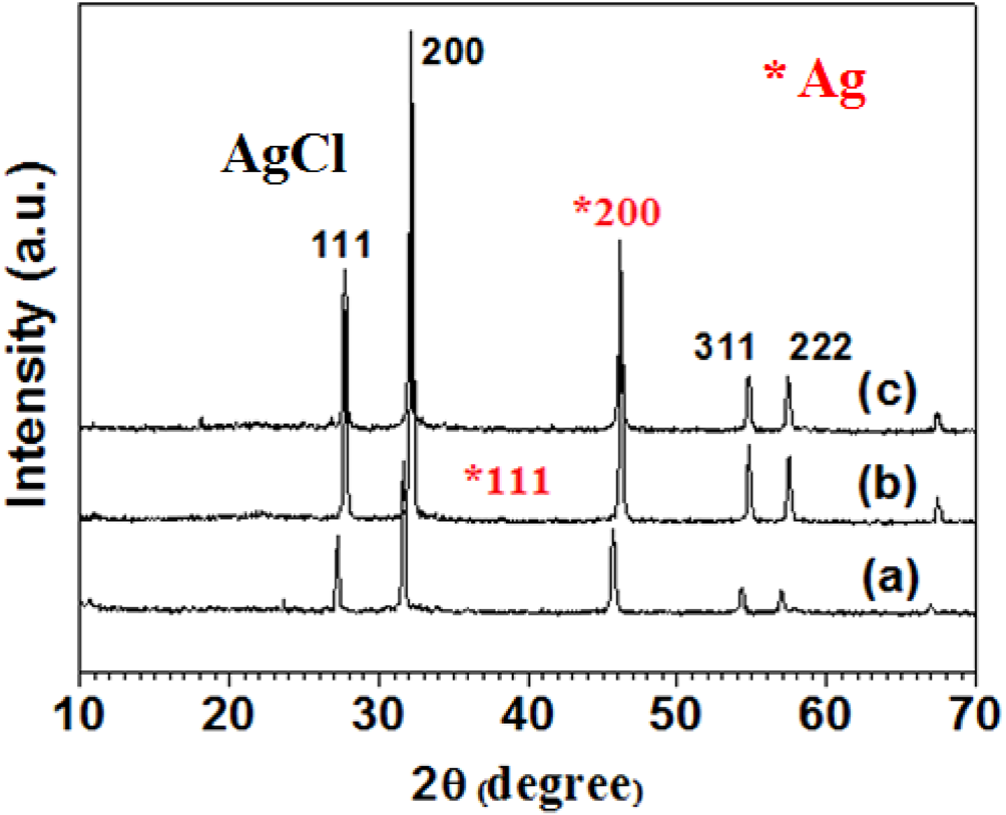
XRD patterns of samples synthesized with 65% (w/w) of ZnCl_2_ solution at different temperatures: (**a**) 50; (**b**) 60; and (**c**) 70 °C, respectively.

The samples were synthesized by green, in-situ method with cellulose solution (dissolution the cellulose in 65% ZnCl_2_ solution) with different temperatures (50, 60 and 70 °C). The corresponding X-ray powder diffraction (XRD) patterns were shown in Fig. 1. According to Fig. 1a–c, all the samples showed similar diffraction peaks. The (111), (200), (311), and (222) planes could be attributed to well-crystallized AgCl with a cubic structure (JCPDS 31-1238), while (200) plane were assigned to crystallized Ag with a cubic structure (marked with, JCPDS 04-0783). Moreover, there is a slight difference for the sample synthesized at 60 °C, a weak peak of (111) plane of crystallized Ag was also observed in Fig. 1b, the densities of Ag peaks become stronger with increasing temperatures was observed in Fig. 1c, implying that the heating temperature can slightly influence the phases of the composites. Some Ag^+^ reacted with Cl^−^ to form AgCl crystals, and some Ag^+^ were reduced to Ag crystals. The peaks of cellulose were not obviously observed, this might be attributed to that the cellulose peaks were overlapped by the strong peak intensities of Ag(AgCl) crystals.

**Figure 2:**
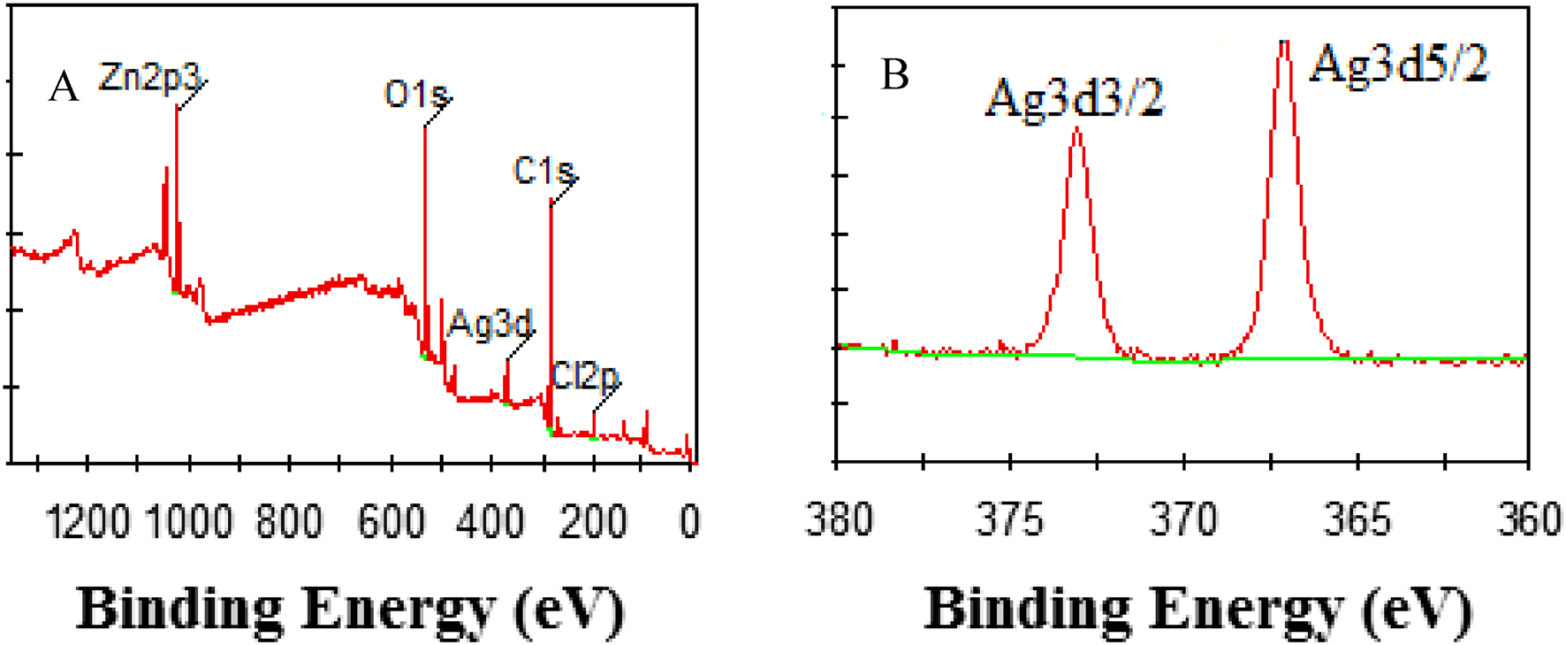
Scanning XPS spectrum (**A**) of the samples synthesized at 60 °C with 65% (w/w) of ZnCl_2_ solution, and the corresponding high-resolution XPS spectra (**B**) of Ag (3d).

In the present work, the chemical compositions and the valence states of the prepared species were further determined by the XPS spectra, as shown in Fig. 2. Figure 2A showed a typical survey-scan XPS spectrum of the sample synthesized at 60 °C with 65% of ZnCl_2_ solution, the Zn, C, O, Ag, and Cl elements were observed clearly. As was shown in Fig. 2B, two peaks were observed in the corresponding high-resolution XPS spectra of Ag 3d orbits. According to the previous work reported by Tang et al.^30^, the peak at 368.0 eV and 373.9 eV can be attributed to the Ag 3d5/2 orbit and Ag 3d3/2 orbit, respectively. Both of the two peaks were attributed to Ag(0), indicating the existence of Ag(0) in the synthesized sample, which is in good accordance with the XRD results in Fig. 1. Based on both of the results from XRD measurement and XPS analysis, one can conclude the existence of Ag@AgCl crystals in the samples synthesized in the present strategy.

**Figure 3:**
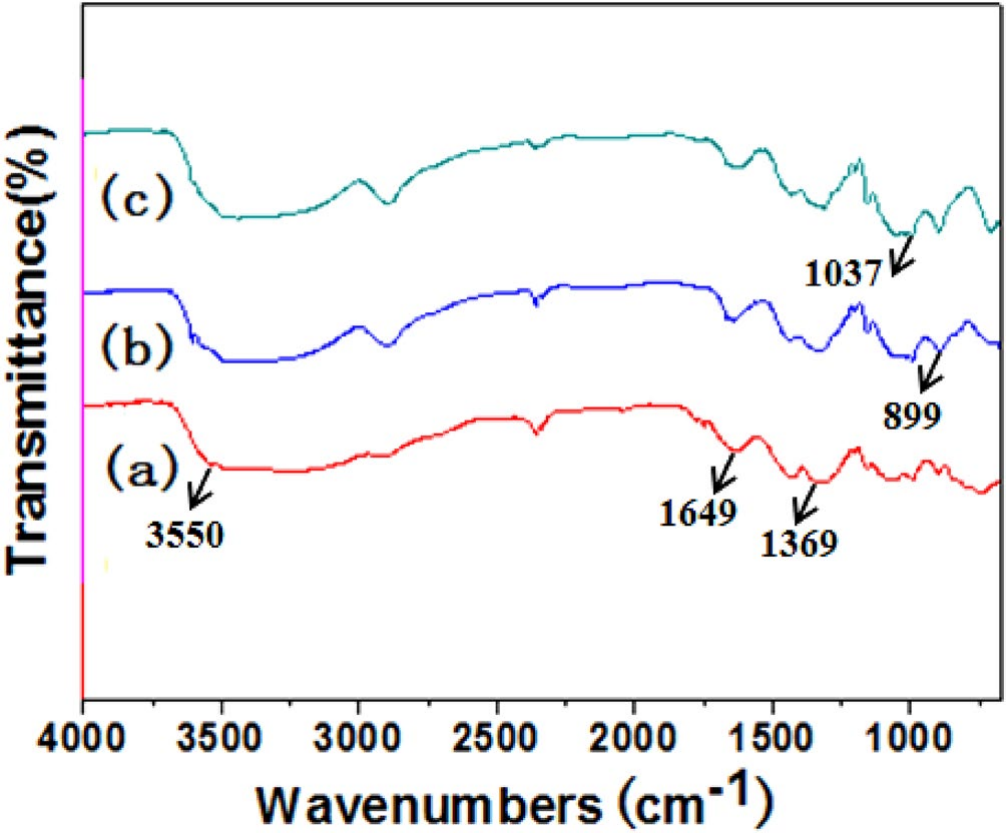
FT-IR spectra of samples synthesized at 60 °C with 65% (w/w) of ZnCl_2_ solution for different times: (**a**) 1 h; (**b**) 2 h and (**c**) 3 h, respectively.

**Figure 4:**
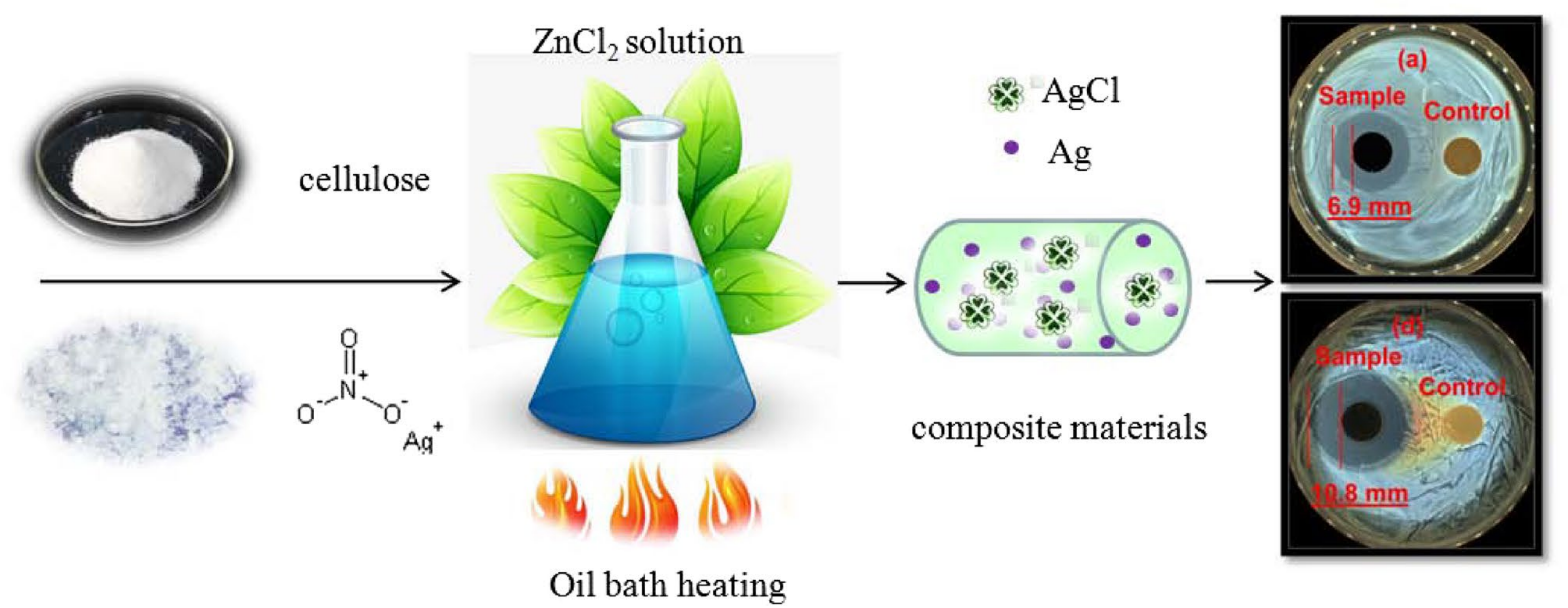
The schematic illustration of the formation process of Ag@AgCl-reinforced cellulose composites in the present strategy. (The illustration is drawn by ourselves).

FTIR analysis is used for studying functional groups of hybrids. The samples were synthesized at 60 °C with 65% (w/w) of ZnCl_2_ solution for 1, 2, and 3 h, respectively, as shown in Fig. 3. All peaks were in accord with the results in the literature^31^. The peaks at 899, 1037, 1369, and 1649 cm^−1^ are assigned to the characteristic of ß-glycosidic linkages, the C-O in cellulose, the O–H bending, and the bending mode of adsorbed water, respectively. Figure 3a–c showed similar FTIR spectra, which displayed the characteristics of cellulose, indicating the existence of cellulose in the as-prepared samples. According to the results from XRD measurement, XPS spectrum and FTIR analysis, one can conclude that Ag@AgCl-reinforced cellulose composites were successfully synthesized in the present strategy. As was shown in Fig. 4, the reduction of Ag ions and the deposition of inorganic particles on cellulose were simultaneously occurred with the dissolution process of cellulose in ZnCl_2_ solution via oil bath heating method.

**Figure 5:**
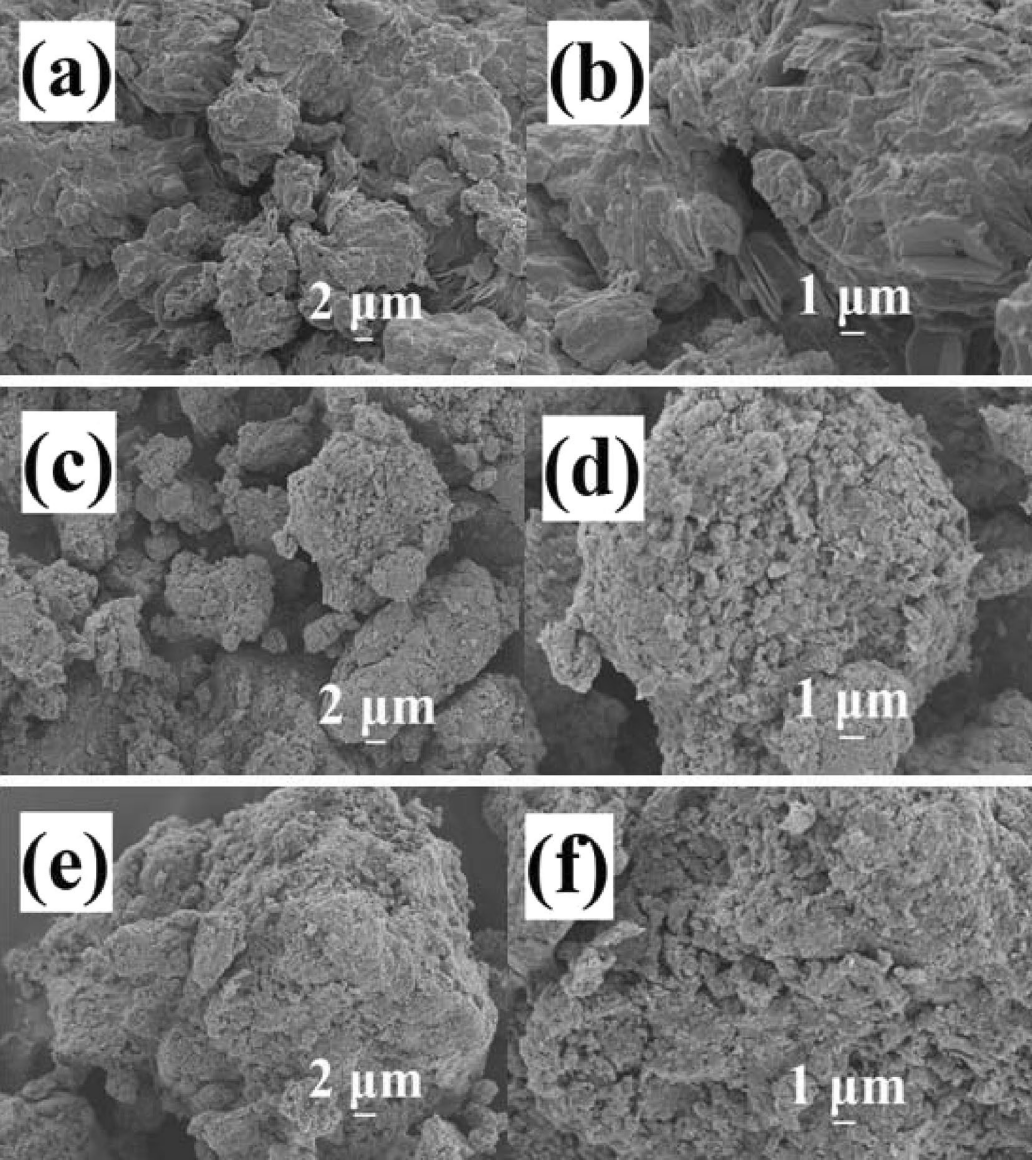
SEM images of the samples synthesized at 60 °C with different concentrations of ZnCl_2_: (**a**,**b**) 60%; (**c**,**d**) 65%; (**e**,**f**) 70%, respectively.

Scanning electron microscope (SEM) was used to characterize the microstructures and morphologies of the as-synthesized samples. Figure 5 showed the SEM images of samples synthesized by oil bath heating method at 60 °C with different concentrations of ZnCl_2_ (60% w/w), ZnCl_2_ (65% w/w), ZnCl_2_ (70% w/w) solutions, respectively. As shown in Fig. 5, the samples synthesized with different concentrations of ZnCl_2_ solutions displayed different morphologies. For the sample synthesized with ZnCl_2_ (60% w/w, Fig. 5a,b), the cellulose displayed bulk structure and clean surface, no obvious aggregation was observed. For the sample synthesized with ZnCl_2_ (65% w/w, Fig. 5c,d), the surface of bulky cellulose became rough, the cellulose was wrapped by a lot of aggregations. Similar morphologies were observed for sample synthesized with ZnCl_2_ (70% w/w, Fig. 5e,f), the surfaces of cellulose were strict wrapped by large amounts of aggregations. Combined with the XRD results in Fig. 1, these aggregations should be assigned to Ag@AgCl crystals. The results of SEM images indicated that higher concentrations of ZnCl_2_ solution favored for the synthesis of Ag@AgCl crystals.

**Figure 6:**
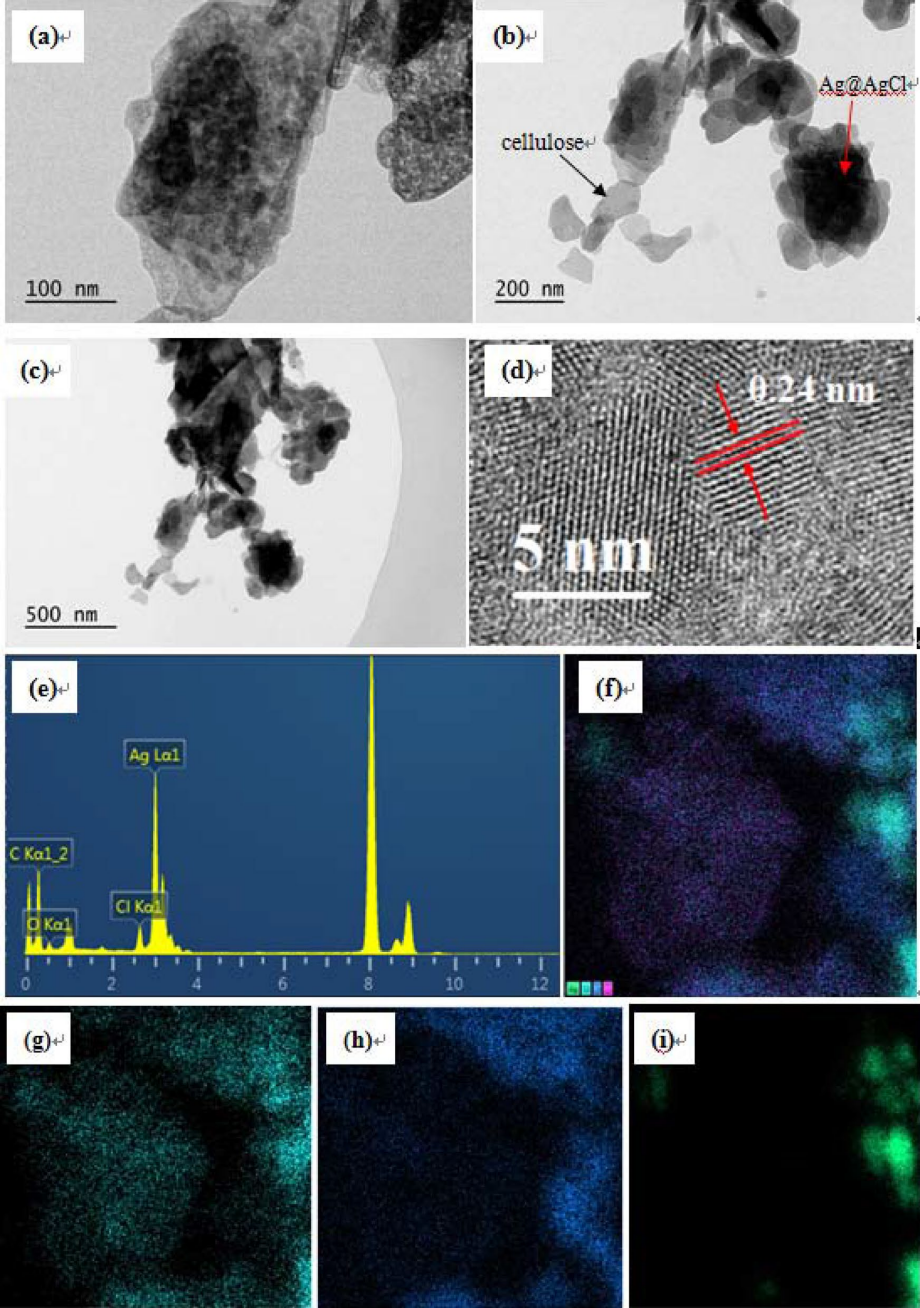
TEM images (**a**–**c**), HR-TEM image (**d**), the corresponding EDS spectrum (**e**), and mapping analysis of existed all elements (**f**), Cl (**g**), C (**h**), Ag (**i**) of the samples synthesized at 60 °C with 65% (w/w) of ZnCl_2_ solution.

The microstructures of Ag@AgCl-reinforced cellulose composites in-situ synthesized via oil bath heating method at 60 °C with 65% of ZnCl_2_ solution were further characterized with transmission electron microscopy (TEM) and high-resolution transmission electron microscopy (HR-TEM), From Fig. 6a–c, one can observe that the inorganic particles (Ag@AgCl) with irregular shapes dispersed in the cellulose matrix. HR-TEM image displayed the lattice spacing determined to be 0.24 nm, which was indexed to the d spacing of the Ag (111) plane (Fig. 6d). From the energy dispersive X-ray spectroscopy (EDS) spectrum in Fig. 6e, one can see that all the peaks were attributed to Ag, C, O, and Cl, which indicated the existence of the Ag, AgCl and cellulose. The mapping analysis of existed elements were showed in Fig. 6f–i. Both the HR-TEM and EDS results further confirmed the successful synthesis of Ag(0) during the present strategy.

**Figure 7:**
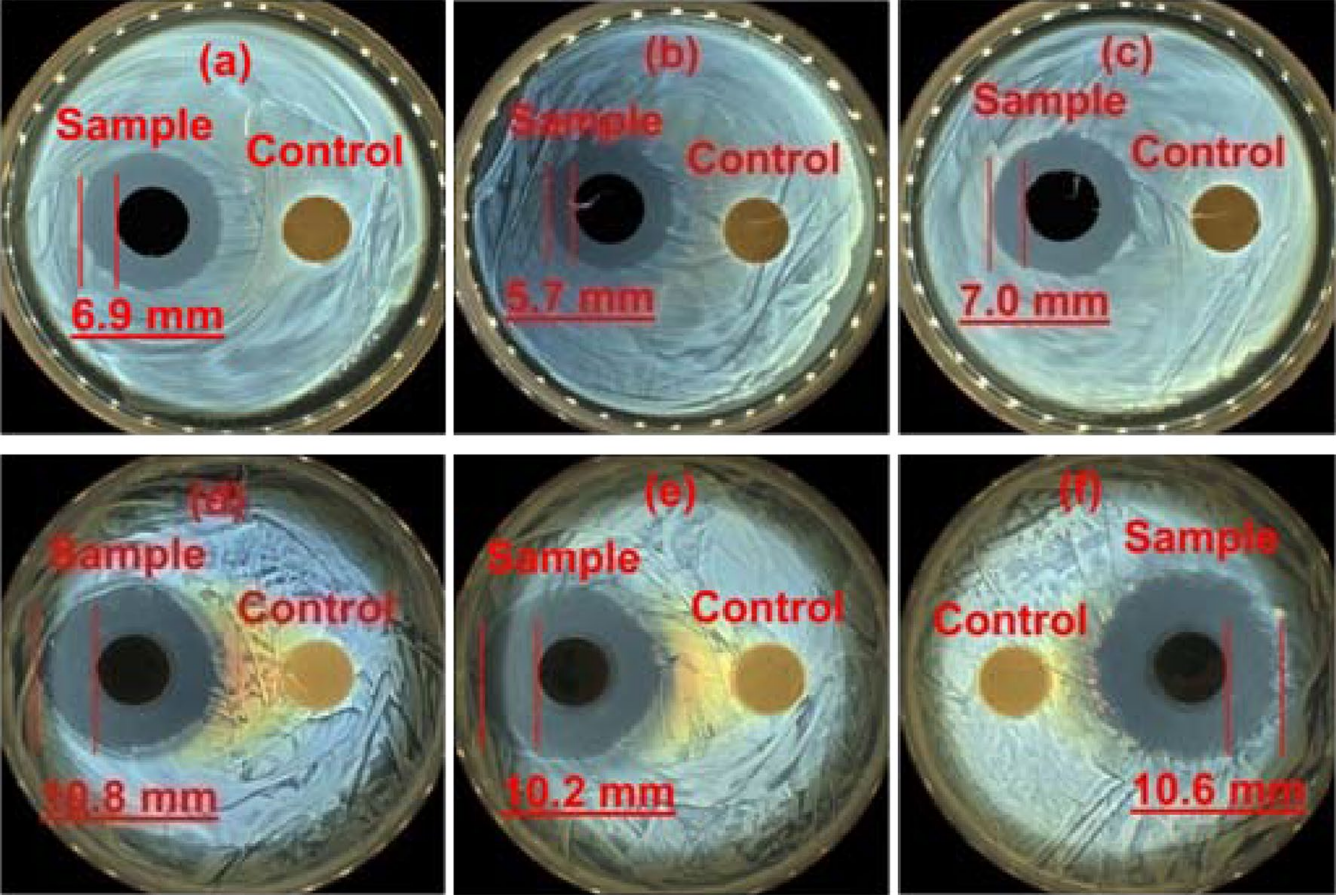
Antibacterial activities of samples synthesized with different concentrations of (**a**,**d**) ZnCl_2_ (65% w/w); (**b**,**c**,**e**,**f**) ZnCl_2_ (70% w/w); (**a**,**b**,**d**,**e**) 0.05 g/0.05 g (samples/MCC); (**c**,**f**) 0.10 g/0.10 g (samples/MCC); (**a**–**c**) for *E. coli*; (**d**–**f**) for *S. aureus*. (The pictures were taken by ourselves during the antibacterial experiments).

The antibacterial activities of the as-synthesized Ag@AgCl-reinforced cellulose composites are illustrated in Fig. 7. The samples chosen for antibacterial experiments are synthesized at 60 °C with ZnCl_2_ (65% w/w) and ZnCl_2_ (70% w/w) for 1 h. Pure cellulose was used as control, but no inhibition zones were obtained, as shown in Fig. 7, indicating that the pure cellulose did not show any antibacterial activities. Both the two samples showed excellent antibacterial activities. The corresponding inhibition zones were summarized in Table 1. From Fig. 7a,d, one can appreciate that the inhibition zones of the sample synthesized with ZnCl_2_ (65% w/w) for *E. coli* and *S. aureus* were 6.9 mm and 10.8 mm, respectively. In case of samples synthesized with ZnCl_2_ (70% w/w), the influence of sample concentrations on their antibacterial activities was explored. As shown in Fig. 7b,e, the inhibition zones of the sample with low concentration of Ag@AgCl-reinforced cellulose composites (0.05 g) for *E. coli* and *S. aureus* were 5.7 mm and 10.2 mm, respectively. For high concentration of sample (0.10 g), they became 7.0 mm and 10.6 mm, respectively (Fig. 7c,f). These results indicated that lower ZnCl_2_ concentration in the synthetic process and a relatively higher samples concentration favored for enhancement in effective antibacterial effect. This may be due to that higher ZnCl_2_ concentration in the synthetic process lead to serious aggregations and poor distribution of Ag@AgCl crystals, which will reduce their antibacterial activities.

**Table 1:** The inhibition zones of Ag@AgCl-reinforced cellulose composites towards both *E. coli* and *S. aureaus*.

ZnCl_2_ solution (w/w) (%)	Sample/cellulose (g/g)	*E. coli* (mm)	*S. aureaus* (mm)
65	(0.05/0.05)	6.9	10.8
70	(0.05/0.05)	5.7	10.2
	(0.10/0.10)	7.0	10.6

**Figure 8:**
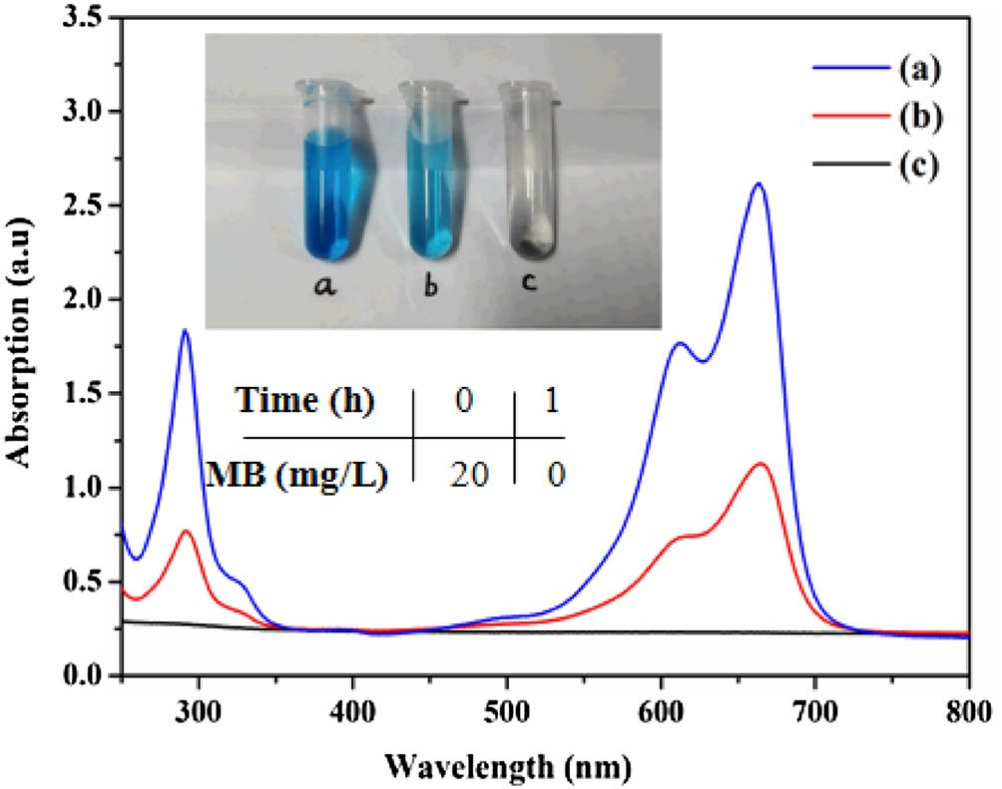
The UV–Visible spectra of methylene *blue* (MB) before and after photocatalytic degradation by Ag@AgCl-reinforced cellulose composites for (**a**) 0, (**b**) 0.5, and 1 h. The up inset is the picture of MB solution before and after photocatalytic degradation by Ag@AgCl-reinforced cellulose composites. The down inset is the MB concentrations with different photocatalytic times.

The photocatalytic activity of the as-prepared Ag@AgCl-reinforced cellulose composites was analyzed by UV–visible spectrum, as was shown in Fig. 8. A kind of azo dyes, methylene blue (MB) was used, which is initially blue, whereas it becomes colorless with degradation of dye molecular. The original concentration of MB was 20 mgL^−1^. The initial MB showed adsorption peak at 664 nm, which was attributed to n–π transition. However, after 0.5 h under UV irradiation, the adsorption intensity of MB solution was decreased obviously, as was shown in Fig. 8b; after 1 h under UV irradiation, one can see that the peaks disappeared at the wavelengths of 664 nm (Fig. 8c), indicating that the as-obtained samples exhibited excellent photocatalytic activity and MB dye could completely degrade in 1 h.

## Conclusions

In summary, Ag@AgCl-reinforced cellulose composites with enhanced antibacterial activities were successfully in-situ synthesized by the oil bath heating method. Experimental results showed that the heating temperature has a slight influence on the phases of the as-synthesized samples. The results of SEM images indicated that Ag@AgCl particles were wrapped on the surface of cellulose, and that higher concentrations of ZnCl_2_ solution favored for the synthesis of Ag@AgCl crystals. Antibacterial activity studies showed that the Ag@AgCl-reinforced cellulose composites exhibited excellent antibacterial activities, the inhibition zones of the samples reach up to 7.0 mm and 10.8 mm against *S. aureus* (Gram-positive bacteria) and *E. coli* (Gram-negative bacteria), respectively. The photocatalytic study indicated that the as-obtained samples exhibited excellent photocatalytic activity and MB dye could completely degrade in 1 h. This synthetic strategy reported here may open a new way to synthesize other functional materials with enhanced properties and opens a new window to high value-added applications of cellulose.
